# Co-endemicity of Pulmonary Tuberculosis and Intestinal Helminth Infection in the People’s Republic of China

**DOI:** 10.1371/journal.pntd.0004580

**Published:** 2016-04-18

**Authors:** Xin-Xu Li, Zhou-Peng Ren, Li-Xia Wang, Hui Zhang, Shi-Wen Jiang, Jia-Xu Chen, Jin-Feng Wang, Xiao-Nong Zhou

**Affiliations:** 1 National Institute of Parasitic Diseases, Chinese Center for Disease Control and Prevention, Shanghai, People’s Republic of China; 2 Key Laboratory of Parasite and Vector Biology, Ministry of Health, WHO Collaborating Centre for Tropical Diseases, Shanghai, People’s Republic of China; 3 National Center for Tuberculosis Control and Prevention, Chinese Center for Disease Control and Prevention, Beijing, People’s Republic of China; 4 State Key Laboratory of Resources and Environmental Information System, Institute of Geographic Sciences and Natural Resources Research, Chinese Academy of Sciences, Beijing, People’s Republic of China; University of Cambridge, UNITED KINGDOM

## Abstract

Both pulmonary tuberculosis (PTB) and intestinal helminth infection (IHI) affect millions of individuals every year in China. However, the national-scale estimation of prevalence predictors and prevalence maps for these diseases, as well as co-endemic relative risk (RR) maps of both diseases’ prevalence are not well developed. There are co-endemic, high prevalence areas of both diseases, whose delimitation is essential for devising effective control strategies. Bayesian geostatistical logistic regression models including socio-economic, climatic, geographical and environmental predictors were fitted separately for active PTB and IHI based on data from the national surveys for PTB and major human parasitic diseases that were completed in 2010 and 2004, respectively. Prevalence maps and co-endemic RR maps were constructed for both diseases by means of Bayesian Kriging model and Bayesian shared component model capable of appraising the fraction of variance of spatial RRs shared by both diseases, and those specific for each one, under an assumption that there are unobserved covariates common to both diseases. Our results indicate that gross domestic product (GDP) per capita had a negative association, while rural regions, the arid and polar zones and elevation had positive association with active PTB prevalence; for the IHI prevalence, GDP per capita and distance to water bodies had a negative association, the equatorial and warm zones and the normalized difference vegetation index had a positive association. Moderate to high prevalence of active PTB and low prevalence of IHI were predicted in western regions, low to moderate prevalence of active PTB and low prevalence of IHI were predicted in north-central regions and the southeast coastal regions, and moderate to high prevalence of active PTB and high prevalence of IHI were predicted in the south-western regions. Thus, co-endemic areas of active PTB and IHI were located in the south-western regions of China, which might be determined by socio-economic factors, such as GDP per capita.

## Author Summary

Pulmonary tuberculosis (PTB) and intestinal helminth infections (IHI) are infectious diseases of poverty, and both diseases affect millions of individuals every year in China. However, a neglected topic for both diseases is their co-endemicity, which mostly occurs in poor areas. This is the first time the co-endemicity of PTB and IHI and their risk factors have been explored by means of a Bayesian geostatistical logistic regression model, a Bayesian Kriging model and a Bayesian shared component model, based on data from the national surveys. Our results indicate that active PTB and IHI prevalence are associated with economic and ecological indices, both individually and collectively, with different disease spectra in different ecosystems. Additionally, we find that the south-western regions of China are the largest clustering areas for prevalence of both diseases, where socio-economic factors, such as GDP per capita may be common risk factors. Both socio-economic factors and epidemiological patterns relevant to control strategies for active PTB and IHI are illustrated clearly in this study, so we have reason to believe that they are essential for devising effective control strategies and should be considered in the control programs for both diseases.

## Introduction

Pulmonary tuberculosis (PTB) and intestinal helminth infection (IHI) are still widespread in China. Both diseases are associated with poverty and both seriously impact on people’s health. The latest national epidemiological survey for PTB was conducted in 2010 and showed that the active PTB prevalence was 459 per 100,000 among those above 15 years old[1]. The latest national survey for major human parasitic diseases, conducted 2001–2004 reported a total of 26 species of helminth with an overall rate of 21.7%. The most common helminth infections found were *Ascaris lumbricoides* (12.5%), hookworm (5.7%), *Trichuris trichiura* (4.2%), *Clonorchis sinensis* (0.6%) and *Taenia* spp. (0.1%)[2].

A syndemic, i.e. an aggregation of two or more diseases in a population, in which there is some level of biological interaction could be at work with respect to PTB and IHI[3]. For instance, there are indications that IHI may be one of the risk factors for the development of active PTB in addition to human immunodeficiency virus infection (HIV)[4] and aggravation of TB was seen after *Opisthorchis* infection[5,6]. Another study reported a possible link between IHI and dysfunction of the protective immune response to Bacillus Calmette Guérin (BCG) vaccine[7]. These findings may have important implications for the strategy to control PTB and IHI in China, which has a high burden of TB and parasitic infections.

There have been only two studies about both diseases in China, one of which provided the prevalence maps of PTB without prevalence predictors, and the other provided the prevalence maps of soil-transmitted helminths using data from different surveys[8,9]. Prevalence predictors and the geographical distributions of these diseases have not been documented at the same time using uniformly-collected data; nor has joint spatial analysis of both diseases been presented. Therefore, it is felt to be essential to explore local variations in active PTB and IHI with the aim of detecting joint clustering of both diseases using the latest national surveys with uniform approaches.

## Materials and Methods

### Ethics statement

This study was approved by the Ethics Review Committee (ERC) of China CDC and ERC of National Institute of Parasitic Diseases, China CDC. All the data were got from databases or yearbooks, not involved in individuals. Therefore, the informed consent was not necessary in this study.

### Data processing

**Fig 1:**
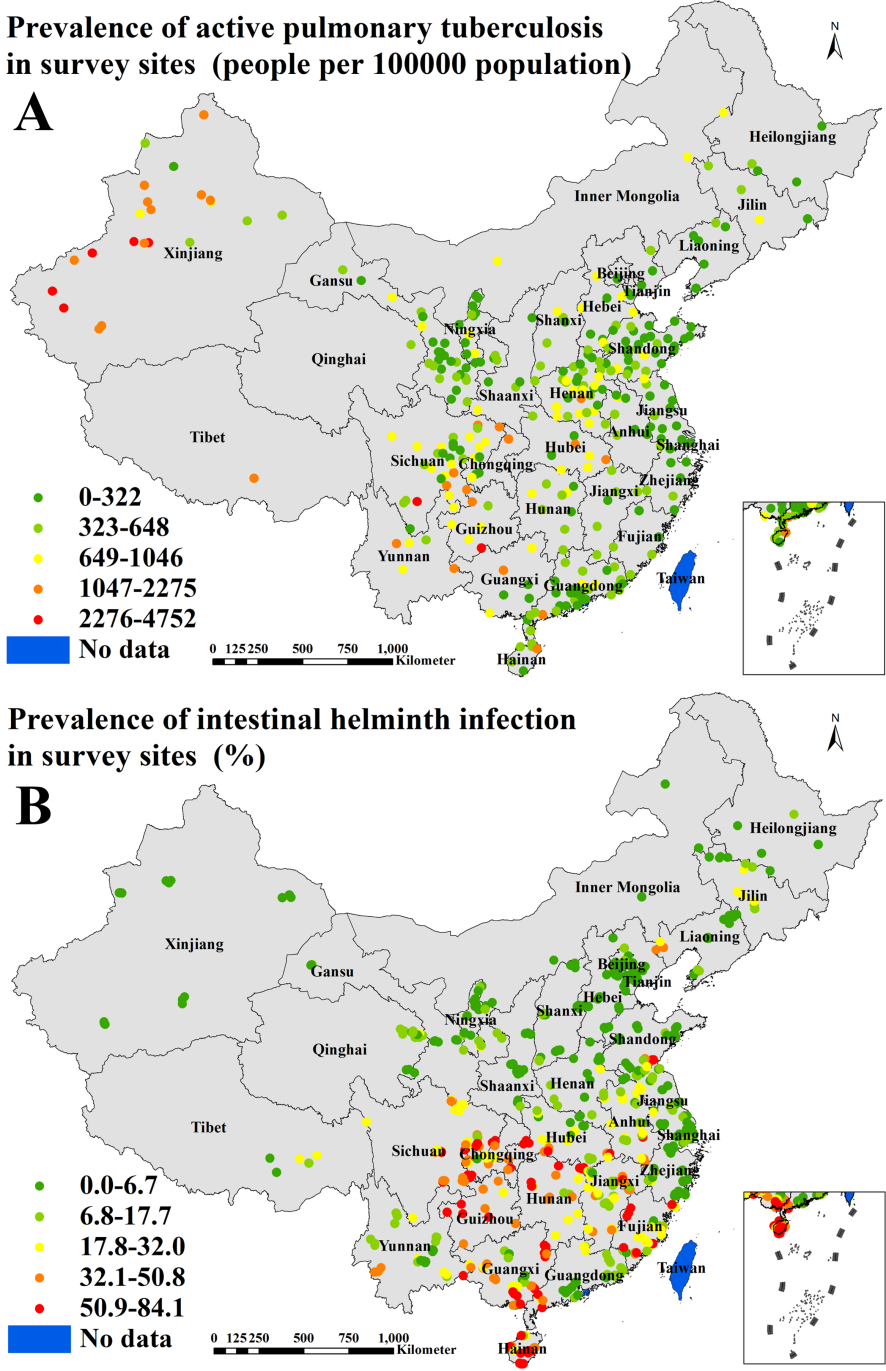
Survey sites and the observed prevalence across P. R. China (A. for active pulmonary tuberculosis; B. for intestinal helminth infection).

The dataset of active PTB prevalence was obtained from the 2010, national TB epidemiological survey[1], which included 176 survey sites with 252,940 participants across the country. In addition to the national survey sites, the provinces of Shandong, Henan, Guangdong, Hainan, Sichuan, Gansu, Ningxia and Xinjiang used the same protocol for extra, provincial survey sites including a total of 151 provincial survey sites with 210,877 participants. Therefore, active PTB prevalence of total 327 survey sites including national and provincial levels were analyzed in this study (Fig 1A). In the survey, chest X-ray was performed on all subjects, and smear microscopy and culture of sputum specimens were carried out on all subjects who showed symptoms of PTB or an abnormal chest X-ray result[1].

Overall prevalence of IHI is infection with any helminth species. The dataset on overall prevalence of IHI was obtained from the national epidemiological survey for major human parasitic diseases conducted from 2001 to 2004[2], which included 687 survey sites with 343,500 participants across the country that were analyzed in this study (Fig 1B). In the survey, the Kato-Katz technique for stool specimens was used to examine the eggs of intestinal helminths, the test tube filter paper culture method was used to identify *Ancylostoma duodenale* and *Necator americanus* and examine other nematode larvae, and the adhesive cellophane anal swab method was used to examine the eggs of *Enterobius vermicularis* and *Taenia* spp. During the fecal examinations, eggs or larvae of other parasites other than the above mentioned parasites were also recorded. The survey showed that major intestinal helminths were *Ascaris lumbricoides*, hookworm, *Trichuris trichiura*, *Clonorchis sinensis* and *Taenia* spp.

**Fig 2:**
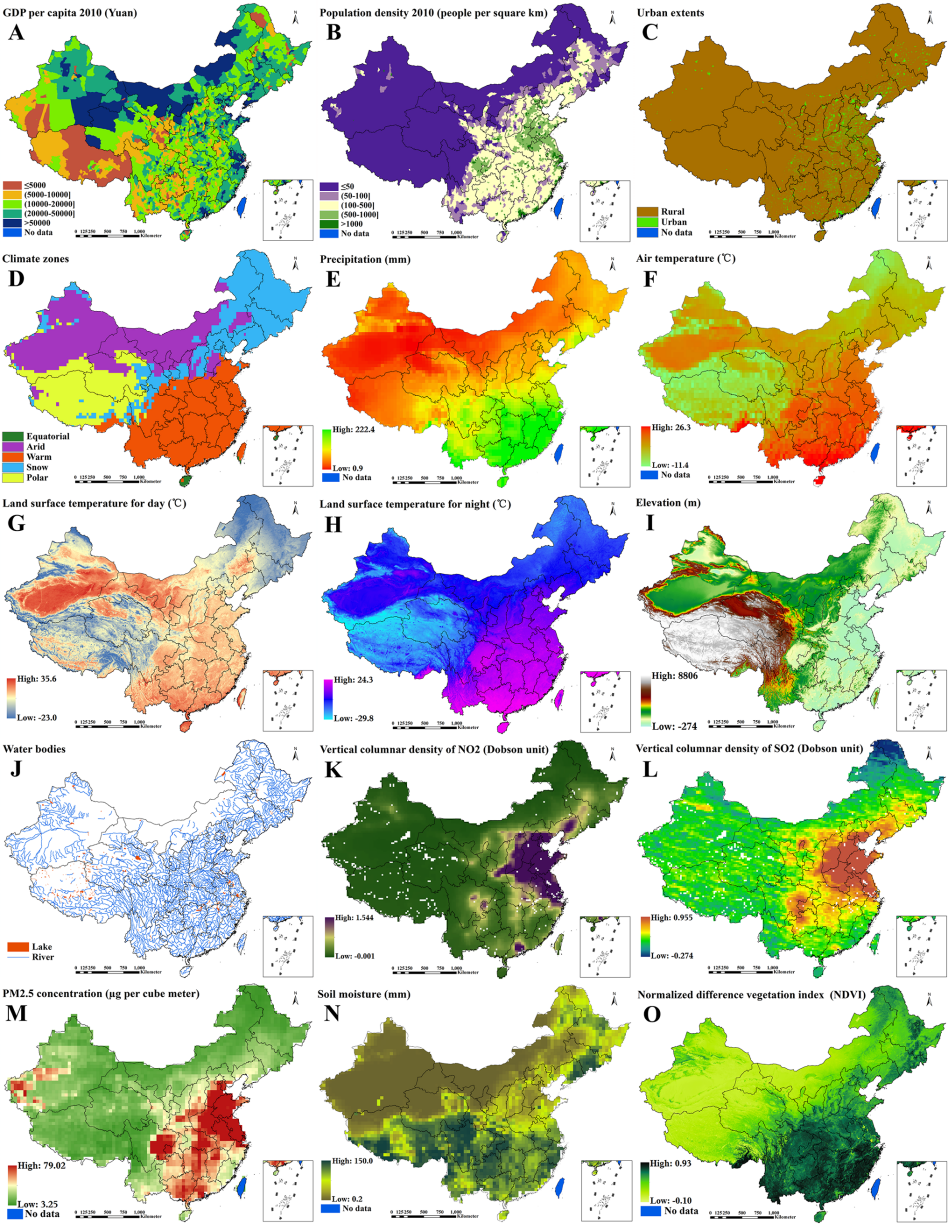
Spatial distributions of covariates across P. R. China (A. gross domestic product [GDP] per capita; B. population density; C. urban extents; D. climate zones; E. precipitation; F. air temperature; G. land surface temperature for day; H. land surface temperature for night; I. elevation; J. water bodies; K. vertical columnar density [VCD] of nitrogen dioxide [NO2]; L. VCD of sulfur dioxide [SO2]; M. concentration of particulate matter of 2.5 micrometers [PM2.5]; N. soil moisture; O. normalized difference vegetation index [NDVI]).

Proxies of socio-economic, climatic, geographical and environmental factors were extracted as covariates from different readily accessible sources, as shown in Tables 1 and 2. The gross domestic product (GDP) per capita, population density and urban extents with a binary indicator of urban/rural extent were included in the analysis to capture influences of social developments and human activities on both diseases[8,10–13]. Climate zones consisting of equatorial, arid, warm, snow and polar zones, precipitation, air temperature and land surface temperature (LST) for day and night were used to reflect impacts of climatic factors on both diseases[8,10,11,14–20], among which air temperature was only included in the analysis of active PTB[19], and LST only in the analysis of IHI[8,11,14–16,18]. Elevation and water bodies were applied to the evaluation of relationships between geographical factors and both diseases[8–11,14–18], among which Euclidean distances from survey sites to water bodies were only included in the analysis of IHI[8,11,14–16]. Vertical columnar density (VCD) of nitrogen dioxide (NO2), VCD of sulfur dioxide (SO2), concentration of particulate matter of 2.5 micrometers (PM2.5), soil moisture and normalized difference vegetation index (NDVI) were used to assess influences of environmental factors on both diseases[8,10,11,14,15,17–19,21,22], among which VCD of NO2, VCD of SO2 and PM2.5 concentration were only included in the analysis of active PTB[19,21,22], and soil moisture and NDVI only in the analysis of IHI[8,10,11,14,15,17,18]. GDP per capita and population density were obtained from the Chinese annual, full-text database, and other data were downloaded from websites providing free geospatial data products. All of the collected covariates for more than one year were averaged. Maps of all covariates can be seen in Fig 2.

**Table 1:** Sources for proxies of socio-economic, climatic, geographical and environmental factors.

Data name	Data period	Temporal resolution	Spatial resolution	Website of data source
GDP per capita	2010	NA	County-level	http://acad.cnki.net/Kns55/brief/result.aspx?dbPrefix=CYFD
Population density	2010	NA	County-level	http://acad.cnki.net/Kns55/brief/result.aspx?dbPrefix=CYFD
Urban extents	1990–2000	NA	1 km	http://sedac.ciesin.columbia.edu/data/set/grump-v1-urban-extents
Climate zones	1976–2000	NA	15km	http://koeppen-geiger.vu-wien.ac.at/shifts.htm
Precipitation	2001–2010	Monthly	50km	http://cdc.cma.gov.cn/dataSetLogger.do?changeFlag=dataLogger
Air temperature	2001–2010	Monthly	50km	http://cdc.cma.gov.cn/dataSetLogger.do?changeFlag=dataLogger
LST for day and night	2001–2010	Monthly	1km	http://www.gscloud.cn/listdata/showinfo_new.shtml?from=&id=336
Elevation	2000	NA	90m	http://www.geodata.cn/Portal/metadata/viewMetadata.jsp?id=100101-11220
Water bodies	2000	NA	NA	http://www.geodata.cn/Portal/metadata/viewMetadata.jsp?id=100101-15
VCD of NO2	2001–2010	Monthly	25km	http://www.geodata.cn/Portal/metadata/viewMetadata.jsp?id=210093-10246
VCD of SO2	2004–2010	Monthly	25km	http://www.geodata.cn/Portal/metadata/viewMetadata.jsp?id=210093-10249
PM2.5 concentration	2001–2010	Annual	50km	http://sedac.ciesin.columbia.edu/data/set/sdei-global-annual-avg-pm2-5-2001-2010
Soil moisture	1950–1999	NA	50km	http://www.sage.wisc.edu/atlas/maps.php?datasetid=23&includerelatedlinks=1&dataset=23
NDVI	2001–2010	Monthly	1km	http://www.gscloud.cn/listdata/showinfo_new.shtml?from=&id=345

GDP, gross domestic product; LST, land surface temperature; VCD, vertical columnar density; NO2, Nitrogen dioxide; SO2, sulfur dioxide; PM2.5, Particulate matter of 2.5 micrometers; NDVI, normalized difference vegetation index; NA, not applicable.

**Table 2:** Overview of prevalence and relevant factors for the survey sites of active pulmonary tuberculosis and intestinal helminth infection.

Variable	Type	Active pulmonary tuberculosis (N = 327)		Intestinal helminth infection (N = 687)	
		Median	Interquartile range	Median	Interquartile range
Prevalence	Continuous	414 / 100,000	222–710 / 100,000	10.0%	2.4–27.9%
GDP per capita (RMB Yuan)	Continuous	18,634	11,718–33,079	19,734	12,366–34,026
Population density (people / km^2^)	Continuous	502	186–1,064	454	172–772
Urban extents^†^	Categorical				
Rural		167	51.10%	437	63.70%
Urban		160	48.90%	250	36.30%
Climate zones^†^	Categorical				
Equatorial		3	0.90%	14	2.00%
Arid		56	17.10%	62	9.00%
Warm		214	65.40%	477	69.50%
Snow		54	16.50%	132	19.20%
Polar		0	0.00%	2	0.30%
Precipitation (mm)	Continuous	64.3	41.5–96.0	80.5	46.3–119.2
Air temperature (°C)	Continuous	14.1	8.7–16.7	NA	NA
LST for day (°C)	Continuous	NA	NA	21.8	20.2–23.8
LST for night (°C)	Continuous	NA	NA	12.3	6.6–14.7
Elevation (m)	Continuous	161	37–848	118	28–546
Distance to water bodies (m)	Continuous	NA	NA	6000	2000–12767
VCD of NO2 (Dobson unit)	Continuous	0.22	0.07–0.56	NA	NA
VCD of SO2 (Dobson unit)	Continuous	0.29	0.16–0.48	NA	NA
PM2.5 concentration (μg / m^3^)	Continuous	32.8	18.8–46.4	NA	NA
Soil moisture (mm)	Continuous	NA	NA	84.0	31.3–113.6
NDVI	Continuous	NA	NA	0.54	0.43–0.64

†, n and %; GDP, gross domestic product; LST, land surface temperature; VCD, vertical columnar density; NO2, Nitrogen dioxide; SO2, sulfur dioxide; PM2.5, Particulate matter of 2.5 micrometers; NDVI, normalized difference vegetation index; NA, not applicable.

All survey sites as well as supporting data for each diseases were converted into feature ESRI datasets (ESRI Inc., Redlands, CA, USA) and then further converted into ESRI raster datasets as needed. All data were processed with ArcGIS 10 (ESRI).

### Bayesian geostatistical logistic regression modeling

The spatial variations in prevalence of active PTB and IHI were modeled using Bayesian geostatistical logistic regression models. The method used is a combination of the logistic regression model and Bayesian Kriging model, which can be used for the analysis of geo-referenced binomial data, e.g., disease prevalence where the outcome variable is bounded between zero and one[23]. The modeling process describes the variability in the outcome variable as a function of the explanatory variables with the addition of a stochastic spatial effect to model the residual spatial autocorrelation. Exponentiation of the model parameters provides the odds ratio (OR) for each covariate which indicates the power and direction of relationships between the explanatory and outcome variables. Detailed descriptions of the structure of the Bayesian geostatistical logistic regression models and the process of model assessment are described in the additional file: S1 Text.

Markov chain Monte Carlo (MCMC) simulation was used to estimate the univariate and multivariate model parameters by geoRglm package of R statistical software (R version 3.0.2, the R Foundation for Statistical Computing). Following a burn-in of 100,000 iterations, the chain was run for a further 500,000 iterations, with every 100^th^ iteration thereafter stored, resulting in a total of 5,000 samples from the posterior distributions, and the convergence was assessed by the Brooks and Roberts diagnostics[24]. The median values from the posterior distribution and their 95% Bayesian credible intervals (CI) were calculated and exponentiated to ORs and their respective uncertainty measures.

Due to convergence and mixing problems when including all of the covariates in the multivariate model, each of the explanatory variables in Tables 1 and 2 was examined independently using a univariate model. All covariates significantly associated with the prevalence of active PTB or IHI (i.e., the 95% Bayesian CI for the OR did not include the value 1) in the univariate model were selected for the multivariate parameter estimation to eliminate the collinearity of covariates. Any covariates that were non-significant in the multivariate model were discarded from the final model through inspection of the regression parameters and 95% Bayesian CIs[25].

We tried many cut-points of each continuous variable in the univariate model to find which cut-point is significant. For example, we tried cut-points of 25%, 50% and 75% to observe the *P* value in the model. If no significance, we continued to try cut-points of 12.5%, 37.5%, 62.5% and 87.5%. If also no significance, we continued to narrow the range. If all the cut-points were no significant, the variable was removed.

Using the geoRglm package of R statistical software, Bayesian Kriging was employed to produce each smooth prevalence map of active PTB and IHI. A 10 ×10 km spatial resolution prediction grid was created at the national-scale, containing covariate values at each prediction location (grid cell). Samples from the predictive distribution for each prediction location were generated using the above MCMC algorithm given the explanatory variables at each grid cell, and the convergence was assessed by the Brooks and Roberts diagnostics[24]. The posterior medians, lower and upper limits of 95% Bayesian CIs, and posterior standard errors from the predictive distributions were extracted to give predicted prevalence and uncertainty estimates at all locations. Based on the predicted prevalence and population density in each grid cell of the smooth prevalence map, we calculated average prevalence of each county and then created a feature ESRI dataset of prevalence by county for both diseases using ArcGIS software. The Natural Breaks (Jenks) method was used to classify the predicted values and their standard errors.

Validation of predicted prevalence of active PTB and IHI was undertaken by randomly sampling 15% of total survey sites as validation set and running the model using the remaining 85% survey sites (training set) and validating the model with the validation set[26]. The accuracy of the prediction was determined in terms of sensitivity and specificity and by the area under curve (AUC) of a receiver-operating characteristic (ROC) curve[27], where the predicted values were compared to the observed values dichotomized at prevalence thresholds of ≥ 20% to assess discriminatory performance of predictions[28]. As a general rule, an AUC between 0.5 and 0.7 indicates a poor discriminative capacity; 0.7–0.9 indicate a reasonable capacity; and > 0.9 indicate a very good capacity[29]. Moreover, we computed the percentage of test locations with the observed disease risk falling inside 95% Bayesian CI of the predicted posterior distribution, and the predictive mean error (ME) between the observed prevalence *π*_*i*_^*obs*^ and the predicted prevalence *π*_*i*_^*pre*^ at location *i*, where *ME* = *∑*_*i = 1*_ (*π*_*i*_^*obs*^ -*π*_*i*_^*pre*^) / *n* (*i* = 1, …, n) [8,17,30].

### Bayesian shared component modeling

Using the GeoBUGS package, version 1.4.3 of the WinBUGS software (Medical Research Council and Imperial College of Science, Technology and Medicine, UK), the above feature ESRI dataset of prevalence by county was used to fit a Bayesian shared component model to jointly analyze the spatial variations of both diseases’ prevalence with common latent risk factors. We assumed that the county-specific relative risks (RRs) of both diseases’ prevalence depend on a shared latent component common to active PTB and IHI, plus additional latent components specific to each disease[31]. These latent components act as surrogates for unmeasured risk factors of prevalence that affect both or only one of the diseases respectively[31]. Detailed descriptions of the structure of the Bayesian shared component model and the process of model assessment are described in the additional file: S2 Text.

Statistical inference of the Bayesian shared component model was made by using the same MCMC algorithm as for the Bayesian geostatistical logistic regression model, and the convergence was assessed using the Brooks and Roberts diagnostics[24]. For this model, the proportion of variability explained by each component for both disease datasets was derived from the empirical variances[32]. The fitting of various models is measured with the deviance information criterion (DIC); the lower the DIC, the better the model fit[33]. Many studies indicated that Bayesian shared component model was superior in terms of goodness of fit, compared with the individual modeling of diseases[32,34–36]. Therefore, we did not compare goodness of fit between the Bayesian shared component model and other relevant models in this study.

## Results

### Data summaries

It can be seen in Table 2 that the median (interquartile range [IQR]) prevalence were 414 / 100,000 (222–710 / 100,000) and 10.0% (2.4–27.9%) for active PTB from 327 survey sites and IHI from 687 survey sites, respectively. The geographical distribution of survey sites and the observed prevalence for each disease are shown in Fig 1. The median (IQR) or proportion of socio-economic, climatic, geographical and environmental covariates for survey sites of both diseases are listed in Table 2 and maps of the spatial distribution of all covariates used in Bayesian geostatistical logistic regression model are provided in Fig 2.

### Univariate parameter estimation

In the univariate Bayesian geostatistical logistic regression model, GDP per capita, population density, urban extents, climate zones, elevation, VCD of NO2 and PM2.5 concentration were significantly correlated with active PTB prevalence, which can be seen in Table 3. Similarly, in the univariate spatial regression model, GDP per capita, urban extents, climate zones, LST for day, LST for night, NDVI and distance to water bodies were significantly correlated with prevalence of IHI, which can be seen in Table 4.

**Table 3:** Posterior summaries (median and 95% Bayesian CI) of the geostatistical model parameters for active pulmonary tuberculosis.

Variable		Estimate of univariate model^†^	Estimate of multivariate model^†^
GDP per capita (RMB Yuan)	≤ 18,400	1.00	1.00
	> 18,400	0.73 (0.62–0.87)	0.82 (0.69–0.98)
Population density (people / km^2^)	≤ 500	1.00	
	> 500	0.67 (0.57–0.81)	
Urban extents	Urban	1.00	1.00
	Rural	1.49 (1.28–1.75)	1.31 (1.08–1.58)
Climate zones	Equatorial, warm temperate & snow	1.00	1.00
	Arid & polar	1.44 (1.08–1.91)	1.32 (1.01–1.74)
Elevation (m)	≤ 100	1.00	1.00
	> 100	1.55 (1.21–1.99)	1.29 (1.02–1.66)
VCD of NO2 (Dobson unit)	≤ 0.19	1.00	
	> 0.19	1.33 (1.03–1.70)	
PM2.5 concentration (μg / m^3^)	≤ 33	1.00	
	> 33	1.39 (1.10–1.76)	
Range (km)		NA	333 (70–842)
Sill		NA	0.92 (0.35–3.50)
Nugget		NA	0.35 (0.29–0.43)

†, regression coefficients are provided as odds ratios; GDP, gross domestic product; VCD, vertical columnar density; NO2, Nitrogen dioxide; PM2.5, Particulate matter of 2.5 micrometers; NA, not applicable.

**Table 4:** Posterior summaries (median and 95% Bayesian CI) of the geostatistical model parameters for intestinal helminth infection.

Variable		Estimate of univariate model^†^	Estimate of multivariate model^†^
GDP per capita (RMB Yuan)	≤ 19,400	1.00	1.00
	> 19,400	0.77 (0.62–0.96)	0.77 (0.62–0.95)
Urban extents	Urban	1.00	
	Rural	1.21 (1.04–1.42)	
Climate zones	Arid, snow & polar	1.00	1.00
	Equatorial & warm	1.68 (1.04–2.63)	1.72 (1.12–2.64)
LST for day (°C)	≤ 24	1.00	
	> 24	1.56 (1.13–2.15)	
LST for night (°C)	≤ 12	1.00	
	> 12	1.55 (1.02–2.39)	
NDVI	≤ 0.61	1.00	1.00
	> 0.61	1.33 (1.12–1.60)	1.24 (1.03–1.52)
Distance to water bodies (m)	≤ 2,000	1.00	1.00
	> 2,000	0.78 (0.63–0.97)	0.78 (0.63–0.95)
Range (km)		NA	328 (164–492)
Sill		NA	3.41 (3.00–4.31)
Nugget		NA	0.34 (0.30–0.43)

†, regression coefficients are provided as odds ratios; GDP, gross domestic product; LST, land surface temperature; NDVI, normalized difference vegetation index.

### Multivariate parameter estimation

In the multivariate Bayesian geostatistical logistic regression model, four variables finally retained significant correlation with active PTB prevalence, which can be seen in Table 3, where GDP per capita > 18,400 RMB Yuan had a protective effect on active PTB prevalence (OR = 0.82 [95% Bayesian CI = 0.69–0.98]), and rural regions, the arid and polar zones and elevation > 100 m had significantly increased risk effects on active PTB prevalence (OR = 1.31 [95% Bayesian CI = 1.08–1.58], OR = 1.32 [95% Bayesian CI = 1.01–1.74] and OR = 1.29 [95% Bayesian CI = 1.02–1.66], respectively).

Similarly, in the multivariate spatial regression model, four variables finally retained significant correlation with prevalence of IHI, which can be seen in Table 4, where GDP per capita > 19,400 RMB Yuan had a protective effect on prevalence of IHI (OR = 0.77 [95% Bayesian CI = 0.62–0.95]), as did distance to water bodies > 2,000 m (OR = 0.78 [95% Bayesian CI = 0.63–0.95]), and the equatorial and warm zones and NDVI > 0.61 had significantly increased risk effects on prevalence of IHI (OR = 1.72 [95% Bayesian CI = 1.12–2.64] and OR = 1.24 [95% Bayesian CI = 1.03–1.52], respectively).

### Model validation results

For Bayesian geostatistical logistic regression models, an AUC for predicting active PTB prevalence was 0.79 (95% CI = 0.65–0.92) and an AUC for predicting prevalence of IHI was 0.87 (95% CI = 0.79–0.96), which indicated a moderately good predictive performance. Moreover, within 95% Bayesian CI, the spatial regression models were able to correctly estimate 84.7% and 94.4% for prevalence of active PTB and IHI, respectively. The MEs for predictive prevalence of active PTB and IHI were 90 / 100,000 and 1.1% respectively, which suggested that the models slightly underestimated prevalence of active PTB and IHI.

### Spatial predictions

**Fig 3:**
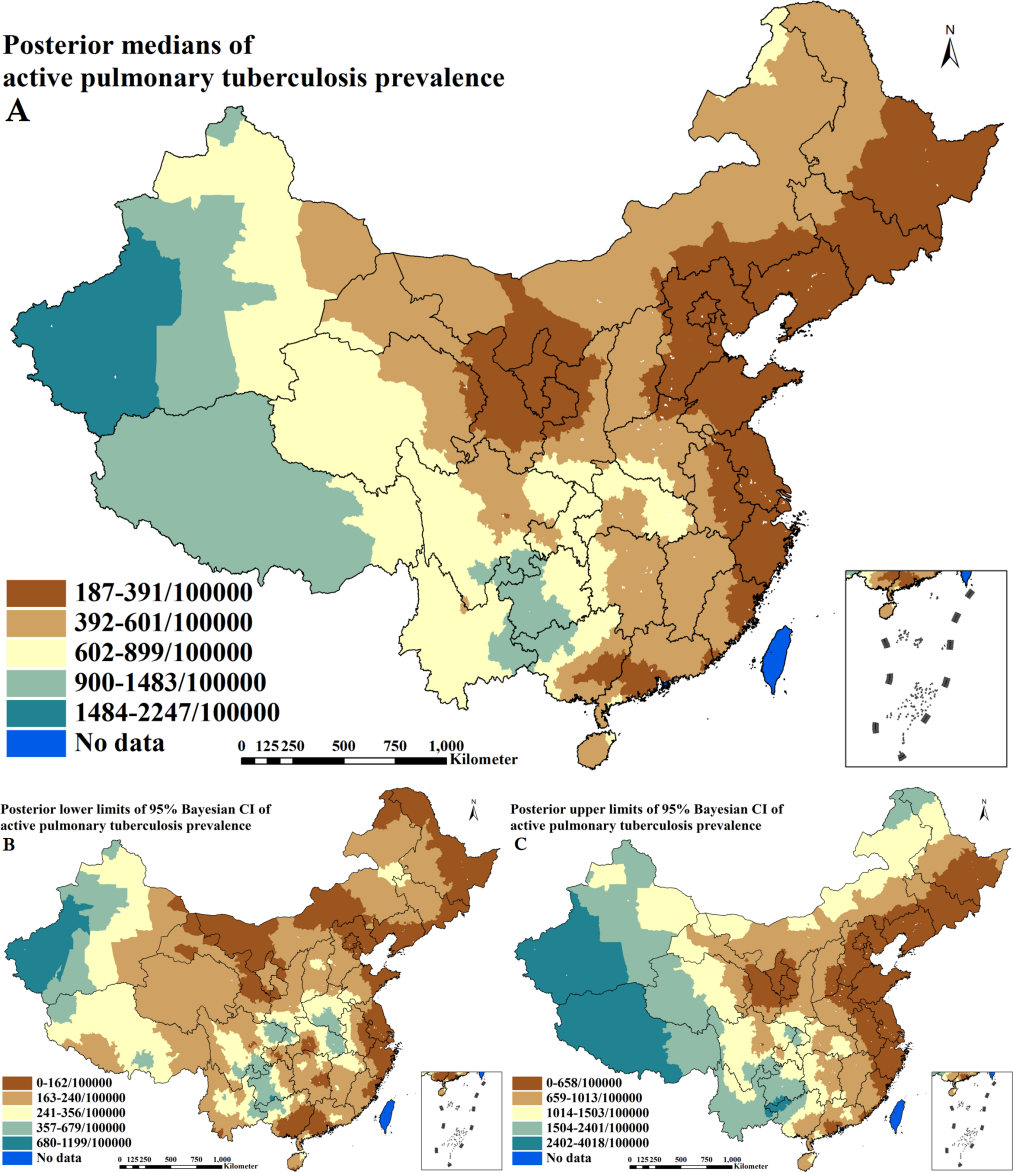
Spatial distributions of active pulmonary tuberculosis across P. R. China (A. posterior medians of prevalence; B. posterior lower limits of 95% Bayesian credible intervals [CI] of prevalence; C. posterior upper limits of 95% Bayesian CI of prevalence).

The predicted prevalence surface of active PTB from the final spatial regression model is illustrated in Fig 3A, 3B and 3C illustrate the lower and upper limits of 95% Bayesian CI for the prediction. High prevalence of active PTB (≥ 900 / 100,000) was predicted in large areas of two provinces including Tibet and Xinjiang and the juncture of four provinces including Guangxi, Sichuan, Guizhou and Yunnan. Low prevalence (≤ 391/100,000) was predicted in most of the south-eastern coastal-line areas, eastern areas of three provinces including Liaoning, Jilin and Heilongjiang, and the juncture of four provinces including Inner Mongolia, Shaanxi, Gansu and Ningxia. Moderate prevalence (392-899/100,000) was predicted between areas of low and high prevalence.

**Fig 4:**
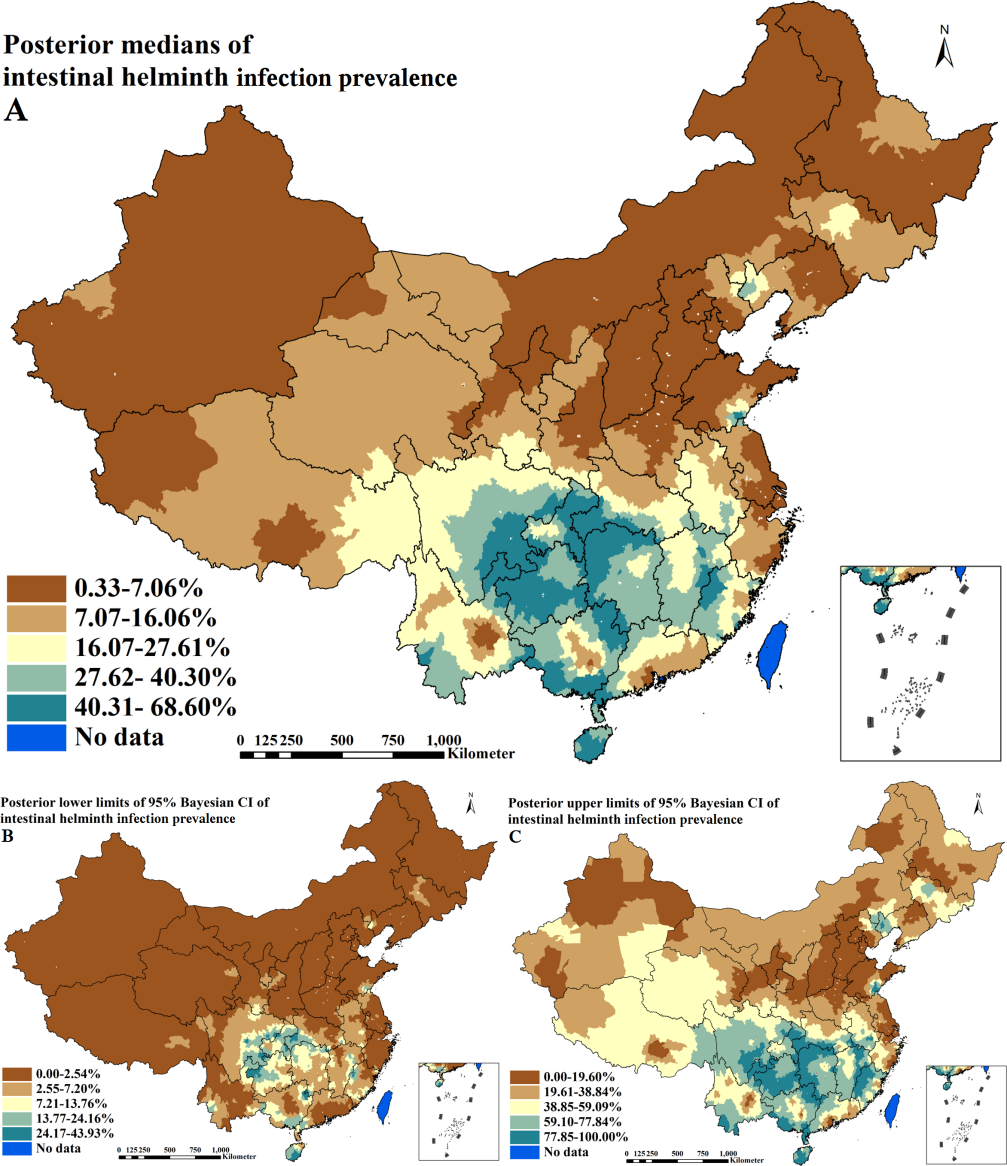
Spatial distributions of intestinal helminth infection across P. R. China (A. posterior medians of prevalence; B. posterior lower limits of 95% Bayesian credible intervals [CI] of prevalence; C. posterior upper limits of 95% Bayesian CI of prevalence).

Similarly, the predicted prevalence surface of IHI from the final spatial regression model is illustrated in Fig 4A, 4B and 4C illustrate the lower and upper limits of 95% Bayesian CI for the prediction. High prevalence of IHI (≥ 27.62%) was predicted in large areas of nine provinces including Fujian, Jiangxi, Hubei, Hunan, Guangxi, Hainan, Chongqing, Sichuan and Guizhou. Low prevalence (≤ 7.06%) was predicted in large areas of 11 provinces including Beijing, Tianjin, Hebei, Shanxi, Inner Mongolia, Liaoning, Shanghai, Jiangsu, Shandong, Henan and Xinjiang. Moderate prevalence (7.07–27.61%) was predicted between areas of low and high prevalence.

For prevalence of both active PTB and IHI, the high prediction uncertainties were correlated with high prevalence areas, which can be seen in the additional file: S1A and S1B Fig, respectively.

### Shared component estimation of relative risks

**Fig 5:**
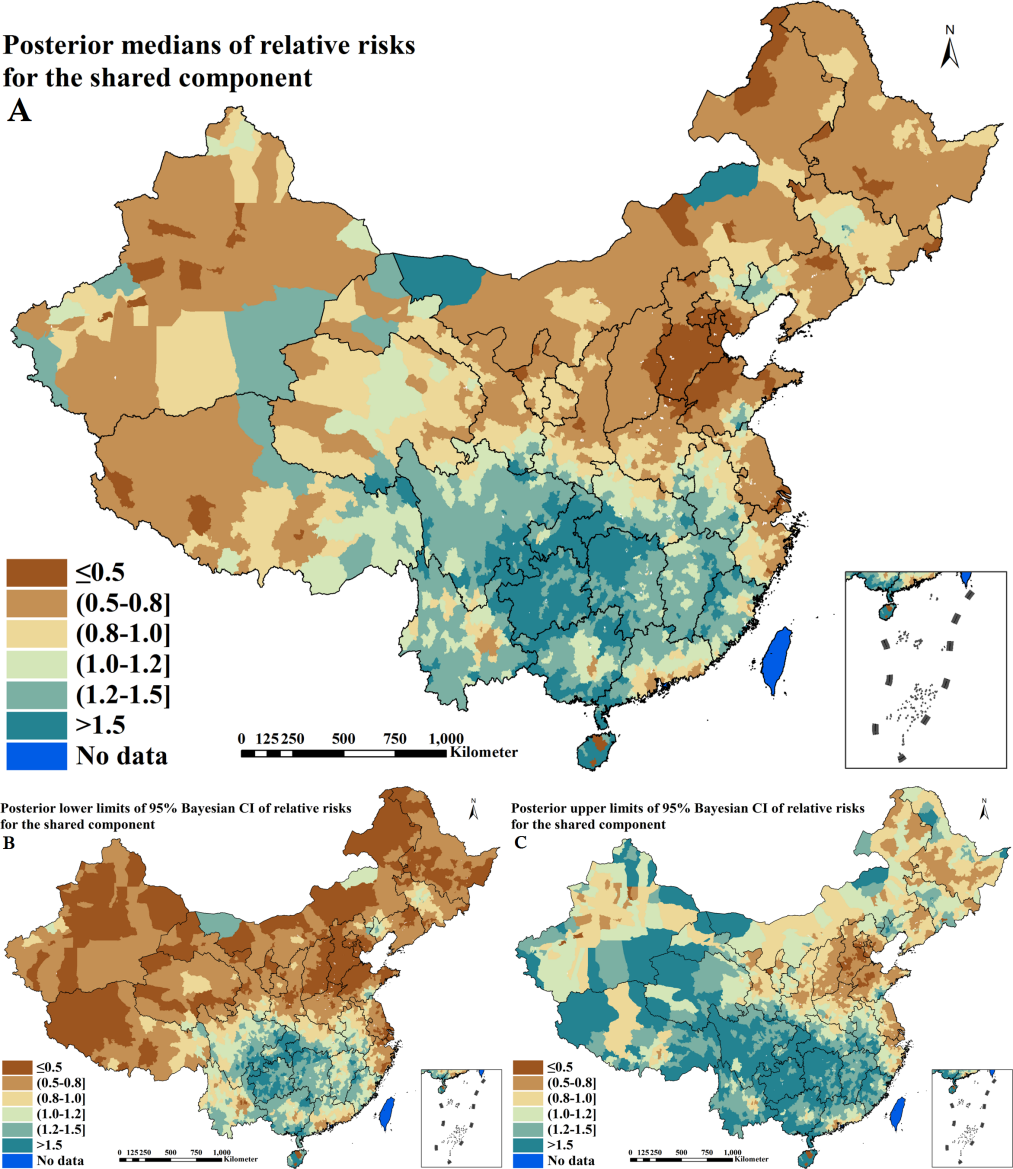
Spatial distributions of the shared component between active pulmonary tuberculosis and intestinal helminth infection across P. R. China (A. posterior medians of relative risks; B. posterior lower limits of 95% Bayesian credible intervals [CI] of relative risks; C. posterior upper limits of 95% Bayesian CI of relative risks)

The shared component of RRs for active PTB and IHI derived from Bayesian shared component model is shown in Table 5 and Fig 5. The shared term captured 28.8% (95% CI = 26.5–30.9%) of the total spatial variation in active PTB, among which 75.1% (95% CI = 63.0–81.2%) was spatially correlated. The shared term captured 69.9% (95% CI = 63.9–74.5%) of the total spatial variation in IHI, among which the same proportion as active PTB (75.1% [95% CI = 63.0–81.2%]) was spatially correlated. Most striking is a large cluster with higher estimation of the shared component (RR > 1.0) in 12 provinces including Anhui, Fujian, Jiangxi, Hubei, Hunan, Guangdong, Guangxi, Hainan, Chongqing, Sichuan, Guizhou and Yunnan. The prediction uncertainties of shared component can be seen in the additional file: S2A Fig.

**Table 5:** Posterior summaries (median and 95% Bayesian CI) of the shared component model parameters by disease.

Parameter	Active pulmonary tuberculosis	Intestinal helminth infection
Variance components		
Shared component	0.068 (0.059–0.076)	0.577 (0.536–0.605)
Unstructured	0.014 (0.011–0.020)	0.123 (0.101–0.151)
Spatial	0.043 (0.033–0.048)	0.370 (0.256–0.438)
Specific component	0.167 (0.163–0.172)	0.250 (0.203–0.304)
Unstructured	0.000 (0.000–0.000)	0.041 (0.038–0.046)
Spatial	0.167 (0.162–0.172)	0.207 (0.123–0.270)
Fraction of total variations		
%Shared component	28.8 (26.5–30.9)	69.9 (63.9–74.5)
%Unstructured	24.9 (18.8–37.0)	24.9 (18.8–37.0)
%Spatial	75.1 (63.0–81.2)	75.1 (63.0–81.2)
%Specific component	71.2 (69.1–73.5)	30.1 (25.5–36.1)
%Unstructured	0.1 (0.0–0.2)	16.3 (13.3–26.9)
%Spatial	99.9 (99.8–100.0)	83.7 (73.1–86.7)

### Disease-specific components estimation of relative risks

**Fig 6:**
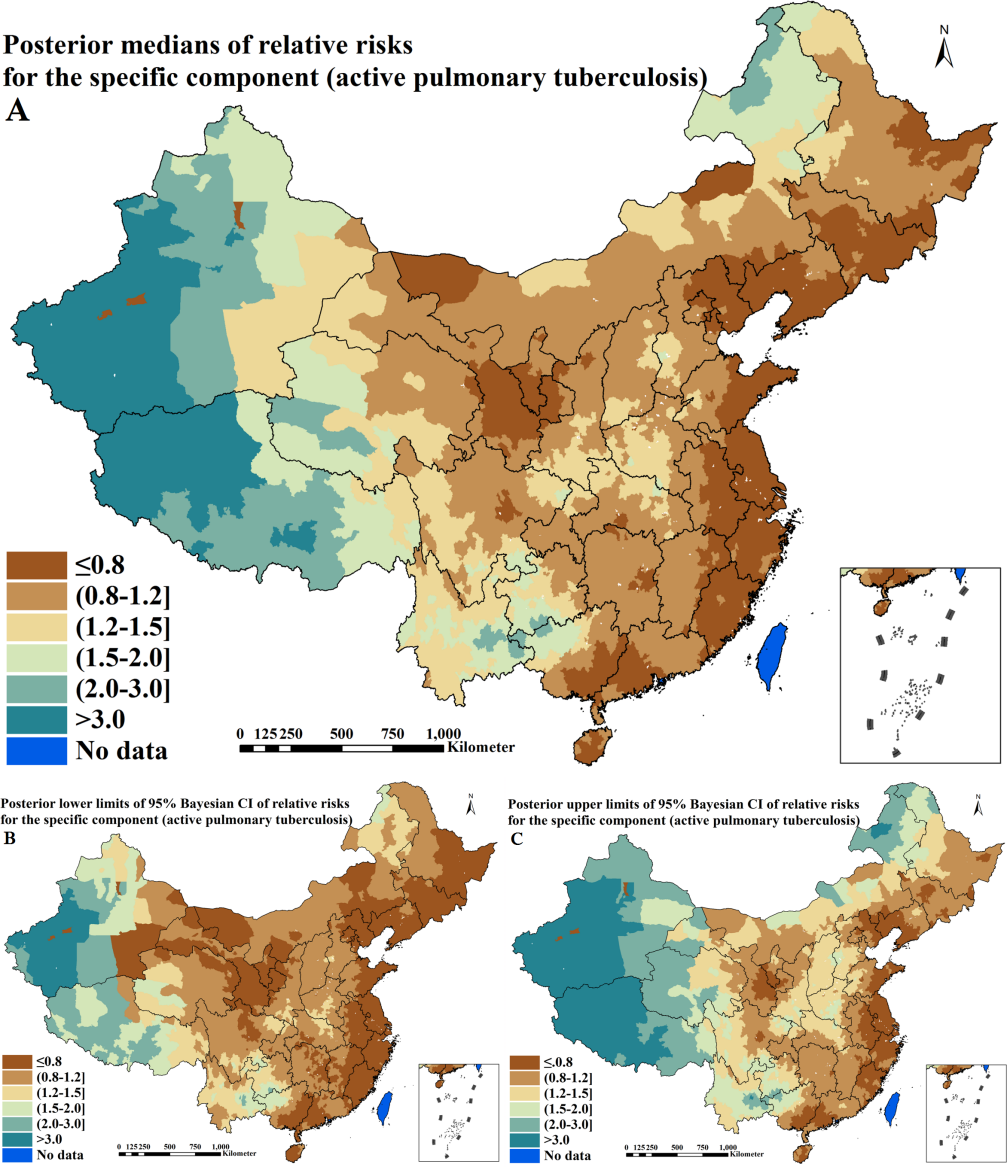
Spatial distributions of the specific component for active pulmonary tuberculosis across P. R. China (A. posterior medians of relative risks; B. posterior lower limits of 95% Bayesian credible intervals [CI] of relative risks; C. posterior upper limits of 95% Bayesian CI of relative risks)

**Fig 7:**
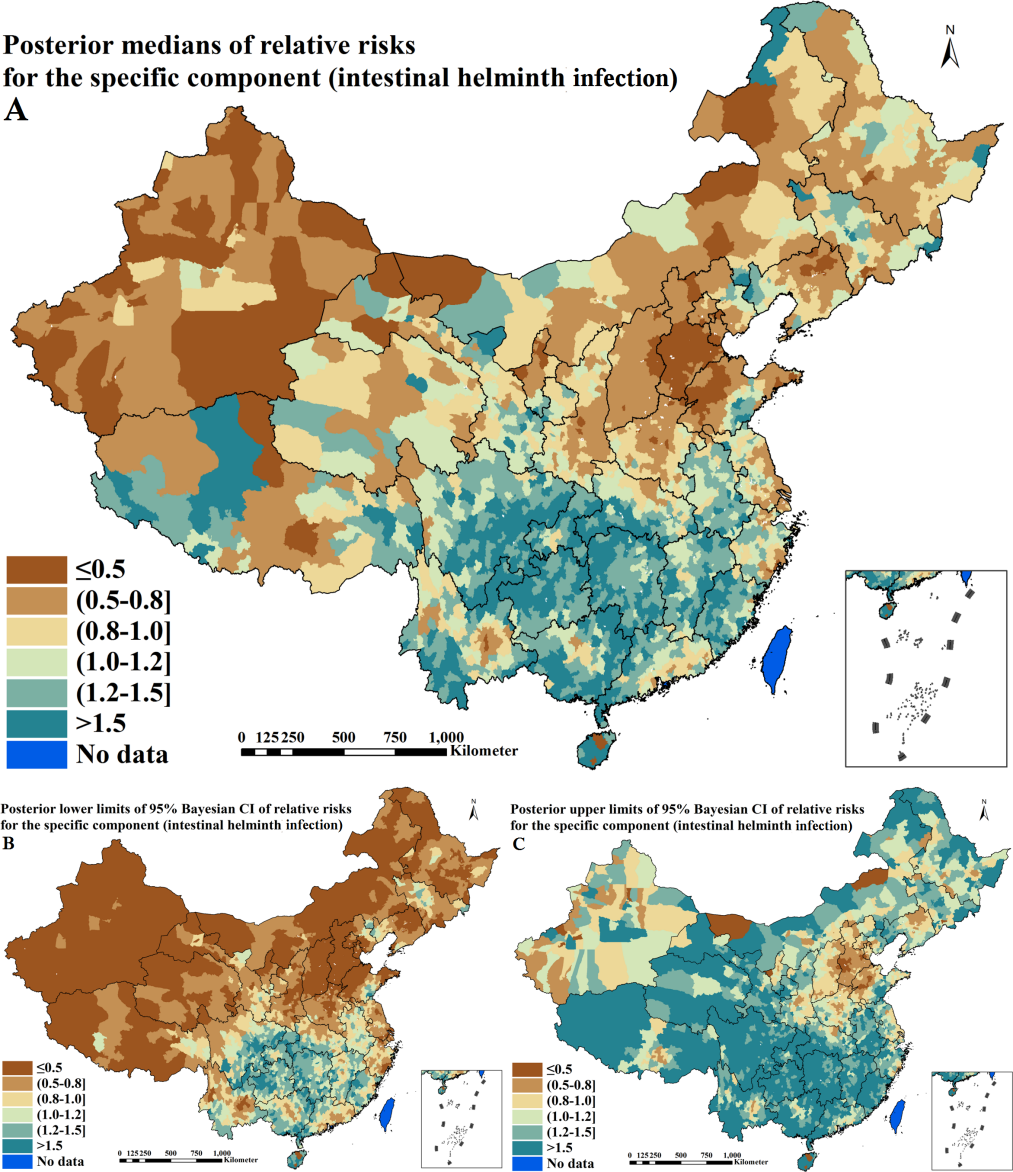
Spatial distributions of the specific component for intestinal helminth infection across P. R. China (A. posterior medians of relative risks; B. posterior lower limits of 95% Bayesian credible intervals [CI] of relative risks; C. posterior upper limits of 95% Bayesian CI of relative risks).

The disease-specific components of RRs for active PTB and IHI derived from Bayesian shared component model are shown in Table 5 and Figs 6 and 7. One disease-specific term captured 71.2% (95% CI = 69.1–73.5%) of the total spatial variation in active PTB, among which 99.9% (95% CI = 99.8–100.0%) was spatially correlated. The other captured 30.1% (95% CI = 25.5–36.1%) of the total spatial variation in IHI, among which 83.7% (95% CI = 73.1–86.7%) was spatially correlated. The disease-specific component for active PTB had a distinct spatial pattern with higher estimation (RR > 1.2) in large areas of seven provinces including Guangxi, Sichuan, Guizhou, Yunnan, Tibet, Qinghai and Xinjiang and the juncture of three provinces including Henan, Hunan and Shaanxi, as shown in Fig 6. The disease-specific component for IHI presented a different spatial pattern with higher estimation (RR > 1.0) in large areas of 12 provinces including Anhui, Fujian, Jiangxi, Hubei, Hunan, Guangdong, Guangxi, Hainan, Chongqing, Sichuan, Guizhou and Yunnan and sparse areas of the remaining provinces, as shown in Fig 7. The prediction uncertainties of disease-specific components can be seen in the additional files: S2B and S2C Fig.

## Discussion

Although the control of both TB and IHI have progressed in China[37,38], there still are millions of new cases of each disease every year. Syndemics of active PTB and IHI may significantly inhibit host immune systems, increase antibacterial therapy intolerance and even alter the protective immune response to vaccination against TB[39], underlining the importance of exploring co-endemic areas. However, there are few model-based, nation-wide predictive infection risk maps for active PTB and IHI in China[8,9]. The estimation of prevalence predictors at the national level and presentation of predictive prevalence maps for active PTB and IHI, as well as co-endemic RR maps of both diseases’ prevalence, are new and accurate as the investigations are based on two recent, national surveys using uniform diagnostic approaches[1,2].

Recently, Bayesian geostatistical analysis was extensively applied to the prediction of parasitic diseases, such as schistosomiasis[40–43], malaria[26,30,44–47], leishmaniasis[48], soil-transmitted helminth infections[8,10,11,14–18], lymphatic filariasis[47,49], but so far there are only few applications to the prediction of TB[50–53]. In addition, Bayesian geostatistical methods have almost exclusively been focused on spatial modeling of a single disease. Here, Bayesian geostatistical techniques was shown to support separate and joint spatial analysis of two different infections, i.e. active PTB and IHI. The approach used in our analysis identified important predictors related to active PTB and IHI. Model validation suggested moderately good predictive ability of our final models according to the validation results that AUCs of prediction were 0.79 and 0.87 and proportions of the observed prevalence correctly predicted within 95% Bayesian CI were 84.7% and 94.4% for active PTB and IHI, respectively. Our final models demonstrated similar, superior predictive performance compared to other studies[8,10,14,16–18]. The MEs (90 / 100,000 and 1.1% for active PTB and IHI, respectively) in this study indicated a slight underestimation of prevalence, which had also been observed in other studies[8,14,17,43]. Therefore, we believe that our predictions provided stable and reliable information about the prevalence of both diseases.

Our results indicated that GDP per capita and population density had negative association with active PTB prevalence in the univariate model, while rural regions, arid and polar zones, elevation, VCD of the NO2 and PM2.5 concentrations showed positive associations. No other study presented all these predictors at a time previously[12,19–22]. Although all these predictors showed association with active PTB prevalence in the univariate model, results of the multivariate model showed that only GDP per capita, urban extents, climate zones and elevation still retained association with active PTB prevalence, which may suggest that these four predictors had greater impact on active PTB prevalence than other predictors in China. However, other studies showed that there was a negative correlation between altitude and TB prevalence[54–57]. In this study, the positive correlation between altitude and active PTB prevalence possibly indicated that the risk effects of other factors overwhelmed the protective effect of altitude[58].

It is indisputable that socio-economic development can inhibit the transmission of various diseases including IHI[8,18,42,48] and our findings were consistent with an earlier study in China[8]. We also found that GDP per capita and distance to water bodies had a negative association with prevalence of IHI in the univariate model, while rural regions, the equatorial and warm zones, LST for day and night and NDVI had a positive association. Despite that all these factors are correlated with the prevalence of IHI in the univariate model, only GDP per capita, climate zones, NDVI and distance to water bodies still retained correlation with prevalence of IHI in the final multivariate model. This may suggest that these four factors had a greater impact on the prevalence of IHI than other factors in our study.

Our predictive prevalence maps for active PTB and IHI presented geographical distribution patterns, which were consistent with previously released maps of both diseases[8,9]. Unsurprisingly, there were obviously different geographical distribution patterns of prevalence between active PTB and IHI. For example, moderate to high prevalence of active PTB was predicted in western regions of the country where only low IHI prevalence was predicted; low to moderate prevalence of active PTB was predicted in the more northern parts of China and the south-eastern, coastal regions where low prevalence of IHI was predicted; moderate to high prevalence of active PTB was predicted in the south-western regions where high prevalence of IHI was predicted.

The shared component explains the fraction of total variation in spatial RRs for each disease in the shared component model. In this study, for IHI about 70% of the total between-area variation in risk was captured by the shared component, while for active PTB about 29% of the total between-area variation in risk was captured by the shared component. This suggests that the shared component had a slightly weaker association with risk of active PTB than with risk of IHI. Although there was a difference between the fractions of both diseases, the shared component still represented the joint prevalence. The spatial analysis of joint prevalence of active PTB and IHI showed that a large cluster of both diseases was found to be located in the south-western regions of the country, which was consistent with the overlapping areas of high prevalence based on separate predictive maps of both diseases. These findings proved the accuracy and reliability of the shared component model used in this study.

**Fig 8:**
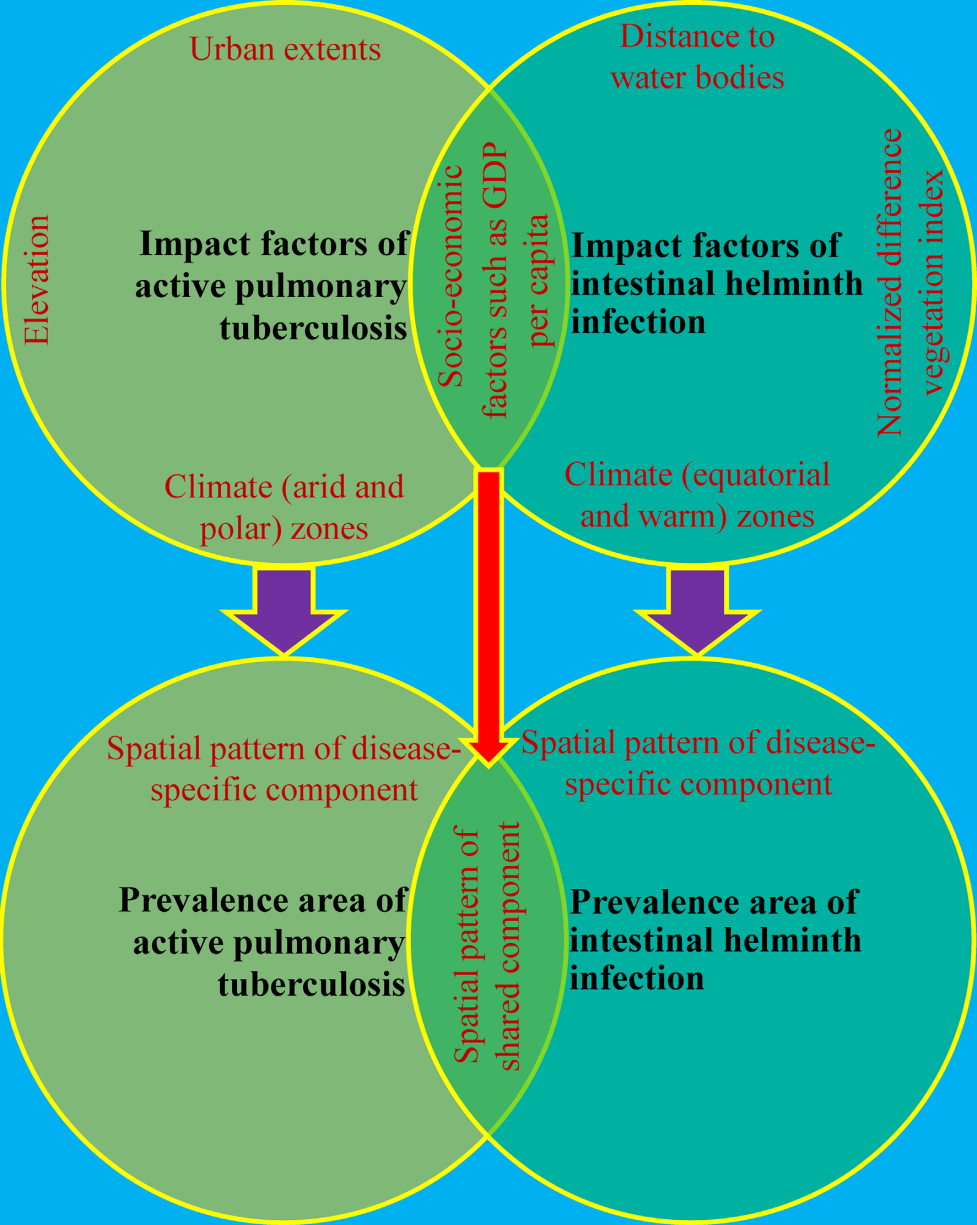
Summarization of relationships between impact factors and spatial patterns of prevalence individually and collectively associated with active pulmonary tuberculosis and intestinal helminth infection in P. R. China.

The shared component model makes the assumption that there are unobserved covariates that display a spatial structure common to both diseases[31]. The analysis results of the separate multivariate model for active PTB and IHI in our study showed that proxies of socio-economic and climatic factors were simultaneously associated with prevalence of both diseases. The socio-economic factors had the same effects on prevalence of both diseases, while the climatic factors showed the opposite effect including positive correlation between the arid and polar zones and active PTB and positive correlation between the equatorial and warm zones and IHI. Therefore, we inferred that socio-economic factors such as GDP per capita were the main unobserved covariates that determined the co-endemic patterns of active PTB and IHI because they were common to both diseases. Moreover, we also observed that the spatial pattern of disease-specific component for active PTB were similar to the distribution of urban extents, climate (arid and polar) zones and elevation in maps, which may indicate that they represented additional risk factors only relevant to active PTB but not to IHI. Similarly, the spatial pattern of disease-specific component for IHI were similar to the distribution of climate (equatorial and warm) zones, NDVI and distance to water bodies in maps, which may indicate that these factors were the additional risk factors only relevant to IHI but not to active PTB (see Fig 8).

All the covariates used in this study were extracted from different accessible sources. Hence, the accuracy and spatial resolution were diverse, possibly influencing the capture of disparities of covariates across the country at uniform scale[10]. For example, although precipitation, air temperature, VCD of SO2 and soil moisture were captured in other studies[8,10,17–21], we did not find them in our study. One possible reason is that data quality of these factors affected the capture of the model, while another possible reason is that these factors really had no association with both diseases. Additionally, both diseases and covariates had high heterogeneity at the national-scale, but we did not divide these data into three or more groups to present the variations in the Bayesian geostatistical logistic regression equation due to limitations of computing power. Furthermore, the shared component model assumes that the shared and specific component are independent, which ignores the possibility of interaction between the real covariates[31]. In view of these limitations, although we believe that our findings provide a useful approximation for both diseases, we caution against over-interpretation of our findings.

In conclusion, our study simultaneously provided prevalence predictors and predictive prevalence maps for active PTB and IHI as well as co-endemic RR maps of both diseases’ prevalence at the national scale. We found that co-endemic areas of active PTB and IHI were located in the south-western regions of China, which may be determined by socio-economic factors such as GDP per capita. Moreover, disease-specific distributions of active PTB may be determined by exclusive factors including urban extents, the arid and polar zones and elevation, while disease-specific distributions of IHI may be determined by exclusive factors including the equatorial and warm zones, NDVI and distance to water bodies. We believe that our estimations provide a valuable assessment of separate and co-endemic situations of active PTB and IHI, therefore we hope that this first effort will contribute useful information to plan syndemic control strategies in co-endemic areas. Additionally, the combination of Bayesian geostatistical techniques in this study provided a new avenue for exploring high prevalence areas of multi-disease syndemics and to understand their interactions at the macro-scale.

## Supporting Information

S1 Text Bayesian geostatistical logistic regression modeling. (DOC)

S2 Text Bayesian shared component modeling. (DOC)

S1 Fig Spatial distributions of posterior standard error of prevalence across P. R. China (A. for active pulmonary tuberculosis; B. for intestinal helminth infection).
(TIF)

S2 Fig Spatial distributions of posterior standard error of relative risks across P. R. China (A. for the shared component between active pulmonary tuberculosis and intestinal helminth infection; B. for the specific component for active pulmonary tuberculosis; C. for the specific component for intestinal helminth infection).
(TIF)
