# Supplementary material for: Co-endemicity of Pulmonary Tuberculosis and Intestinal Helminth Infection in the People’s Republic of China
Source: PLoS Negl Trop Dis. 2016 Apr 18;10(4):e0004580. doi: 10.1371/journal.pntd.0004580 (PMC4835095; doi:10.1371/journal.pntd.0004580)
Supplement: S1 Text — (DOC) [file pntd.0004580.s001.doc]

**Co-endemicity of Pulmonary Tuberculosis and Intestinal Helminth Infection in the People’s Republic of China**

**Supplementary A:**

We assumed that the total number of patients (active pulmonary tuberculosis or intestinal helminth infection) *Y_i_* at survey site *i* (*i* = 1, …, n) follows a conditionally independent binomial distribution *Y_i_* *~ Bin* (*p_i_*, *N_i_*), where *N_i_* is the screened individuals and *p_i_* is the underlying population prevalence at survey site *i*. The method is an extension of Bayesian Kriging model using the logit link function, and the influence of covariates *X_ki_* (*k* = 1, …, m), location-specific spatial random effects *ε_i_* and exchangeable non-spatial random effect *φ_i_* are modeled on the logit, as logit (*p_i_*) = *β_0_* + *β_1_X_1i_ +* … + *β_k_X_ki_* + *ε_i_* + *φ_i_*, where *β_k_* is the regression coefficient of the *k^th^* covariate *X_ki_*. We assumed that *ε_i_* follows a zero-mean multivariate normal distribution (latent stationary Gaussian process) *ε_i_ ~ MVN* (0, Σ), where Σ is a matrix with elements Σ*_ij_*. Assuming an isotropic exponential covariance function, the matrix elements Σ*_ij_* are defined by Σ*_ij_* = *σ^2^_sp_* exp (*-d_ij_* / *ρ*), where *σ^2^_sp_* represents the variance of the spatial process (the sill), *d_ij_* is the Euclidean distance between locations *i* and *j, ρ* is parameter of spatial correlation decay with *d_ij_*. The spatial range *κ* = 3*ρ*, is the minimum distance where spatial correlation is less than 5%. We assumed that *φ_i_* follows a zero-mean normal distribution *φ_i_* *~ N* (0, *σ^2^_nonsp_*), where *σ^2^_nonsp_* represents the variance of the non-spatial process (the nugget effect) . A normal prior distribution was assigned to the regression coefficients, that is *β_0_*, *β_k_* *~* (0, 1000) and log *gamma* priors were adopted for the precision parameters, *τ_sp_* = 1 / *σ^2^_sp_* and *τ_nonsp_* = 1 / *σ^2^_nonsp_* on the log scale, that is log (*τ_sp_*) *~* log *gamma* (1, 0.00005) and log (*τ_nonsp_*) *~* log *gamma* (1, 0.00005). Moreover, we assumed the following prior distribution for range parameter log (*κ*) *~* log *gamma* (1, 0.01) .

**References**

1. Diggle PJ, Ribeiro PJ. Model-based Geostatistics. New York: Springer Science + Business Media, LLC; 2007.

2. Lai YS, Zhou XN, Utzinger J, Vounatsou P. Bayesian geostatistical modelling of soil-transmitted helminth survey data in the People's Republic of China. Parasites & vectors. 2013;6:359. Epub 2013/12/20. doi: 10.1186/1756-3305-6-359. PubMed PMID: 24350825; PubMed Central PMCID: PMC3892068.

3. Giardina F, Gosoniu L, Konate L, Diouf MB, Perry R, Gaye O, et al. Estimating the burden of malaria in Senegal: Bayesian zero-inflated binomial geostatistical modeling of the MIS 2008 data. PloS one. 2012;7(3):e32625. Epub 2012/03/10. doi: 10.1371/journal.pone.0032625. PubMed PMID: 22403684; PubMed Central PMCID: PMC3293829.

4. Gosoniu L, Veta AM, Vounatsou P. Bayesian geostatistical modeling of Malaria Indicator Survey data in Angola. PloS one. 2010;5(3):e9322. Epub 2010/03/31. doi: 10.1371/journal.pone.0009322. PubMed PMID: 20351775; PubMed Central PMCID: PMC2843626.

5. Gosoniu L, Msengwa A, Lengeler C, Vounatsou P. Spatially explicit burden estimates of malaria in Tanzania: bayesian geostatistical modeling of the malaria indicator survey data. PloS one. 2012;7(5):e23966. Epub 2012/06/01. doi: 10.1371/journal.pone.0023966. PubMed PMID: 22649486; PubMed Central PMCID: PMC3359352.

6. Slater H, Michael E. Mapping, bayesian geostatistical analysis and spatial prediction of lymphatic filariasis prevalence in Africa. PloS one. 2013;8(8):e71574. Epub 2013/08/21. doi: 10.1371/journal.pone.0071574. PubMed PMID: 23951194; PubMed Central PMCID: PMC3741112.

7. Wardrop NA, Atkinson PM, Gething PW, Fevre EM, Picozzi K, Kakembo AS, et al. Bayesian geostatistical analysis and prediction of Rhodesian human African trypanosomiasis. PLoS neglected tropical diseases. 2010;4(12):e914. Epub 2011/01/05. doi: 10.1371/journal.pntd.0000914. PubMed PMID: 21200429; PubMed Central PMCID: PMC3006141.

8. Chammartin F, Scholte RG, Malone JB, Bavia ME, Nieto P, Utzinger J, et al. Modelling the geographical distribution of soil-transmitted helminth infections in Bolivia. Parasites & vectors. 2013;6:152. Epub 2013/05/28. doi: 10.1186/1756-3305-6-152. PubMed PMID: 23705798; PubMed Central PMCID: PMC3681678.

9. Chammartin F, Hurlimann E, Raso G, N'Goran EK, Utzinger J, Vounatsou P. Statistical methodological issues in mapping historical schistosomiasis survey data. Acta tropica. 2013;128(2):345-52. Epub 2013/05/08. doi: 10.1016/j.actatropica.2013.04.012. PubMed PMID: 23648217.
