# Supplementary material for: Co-endemicity of Pulmonary Tuberculosis and Intestinal Helminth Infection in the People’s Republic of China
Source: PLoS Negl Trop Dis. 2016 Apr 18;10(4):e0004580. doi: 10.1371/journal.pntd.0004580 (PMC4835095; doi:10.1371/journal.pntd.0004580)
Supplement: S2 Text — (DOC) [file pntd.0004580.s002.doc]

**Co-endemicity of Pulmonary Tuberculosis and Intestinal Helminth Infection in the People’s Republic of China**

**Supplementary B:**

Making the infectious disease assumption, the likelihood for each disease is assumed to be conditionally independent binomial distribution, *Y_1i_* *~ Bin* (*p_1i_*, *N_1i_*) and *Y_2i_* *~ Bin* (*p_2i_*, *N_2i_*), log (*p_1i_*) = log (*P_1_*) + *α_1_* + *λ_i_* * *δ* + *ψ_1i_* and log (*p_2i_*) = log (*P_2_*) + *α_2_* + *λ_i_* / *δ* + *ψ_2i_*, where *Y_1i_* and *Y_2i_* are the patients of active pulmonary tuberculosis and intestinal helminth infection respectively, *N_1i_* and *N_2i_* are the population and *p_1i_* and *p_2i_* are the underlying population prevalence in county *i* (*i* = 1, …, n) respectively, *P_1_* and *P_2_* are overall prevalence at the national-scale of both diseases respectively which assumes the same prevalence in each county, *α_1_* and *α_2_* are intercepts representing the baseline (log) relative risks of both diseases across the country respectively, *δ* is a scaling factor to allow the risk gradient associated with the shared component to be different for each disease, *λ_i_* represents the common (shared) component and *ψ_1i_* and *ψ_2i_* represent two disease-specific (residual) components respectively in county *i*. Each of the three components (*λ*, *ψ_1_* and *ψ_2_*) is assumed to be spatially structured with zero mean, and the components are assumed to be independent of each other.

Besag, York and Mollié (BYM) convolution prior distributions was assigned to each component. We assumed the spatial priors for the shared random effects, as *λ_i_* = *μ_λi_* + *ζ_λi_*, where *μ_λi_* is unstructured shared random effects and follows a zero-mean normal distribution *μ_λi_* *~ N* (0, *ω_μ_*) and *ζ_λi_* is spatial shared random effects and follows a conditional autoregressive (CAR) normal distribution *ζ_λi_* *~* *CARNormal* (W, *ω_ζ_*) with W the matrix representing the neighborhood structure (here two counties are assumed as neighbors if they share a common boundary). We also assumed the spatial priors for the disease-specific random effects, as *ψ_i_* (*ψ_1i_* or *ψ_2i_*) *=* *μ_ψi_* + *ζ_ψi_*, where *μ_ψi_* is unstructured disease-specific random effects and follows a zero-mean normal distribution *μ_ψi_* *~ N* (0, *τ_μ_*) and *ζ_ψi_* is spatial disease-specific random effects and follows a CAR normal distribution *ζ_λi_* *~* *CARNormal* (W, *τ_ζ_*). Finally, the hyperprior specifications for the parameters were *α* *~* *dflats* ( ), log (*δ*) *~* *N* (0, 5.9), and *ω_μ_ ~* *gamma* (0.5, 0.0005), *ω_ζ_* *~* *gamma* (0.5, 0.0005), *τ_μ_ ~* *gamma* (0.5, 0.0005) and *τ_ζ_* *~* *gamma* (0.5, 0.0005).

**References**

1. Held L, Graziano G, Frank C, Rue H. Joint spatial analysis of gastrointestinal infectious diseases. Statistical methods in medical research. 2006;15(5):465-80. doi: 10.1177/0962280206071642

2. Lunn DJ, Thomas A, Best N, Spiegelhalter D. WinBUGS - a Bayesian modelling framework: concepts, structure, and extensibility. Statistics and Computing. 2000;10(4):325-37.

3. Besag J, York J, Mollié A. Bayesian image restoration, with two applications in spatial statistics. Annals of the Institute of Statistical Mathematics. 1991;43(1):1-20.
