# Supplementary figures and images for: Co-endemicity of Pulmonary Tuberculosis and Intestinal Helminth Infection in the People’s Republic of China
Source: PLoS Negl Trop Dis. 2016 Apr 18;10(4):e0004580. doi: 10.1371/journal.pntd.0004580 (PMC4835095; doi:10.1371/journal.pntd.0004580)

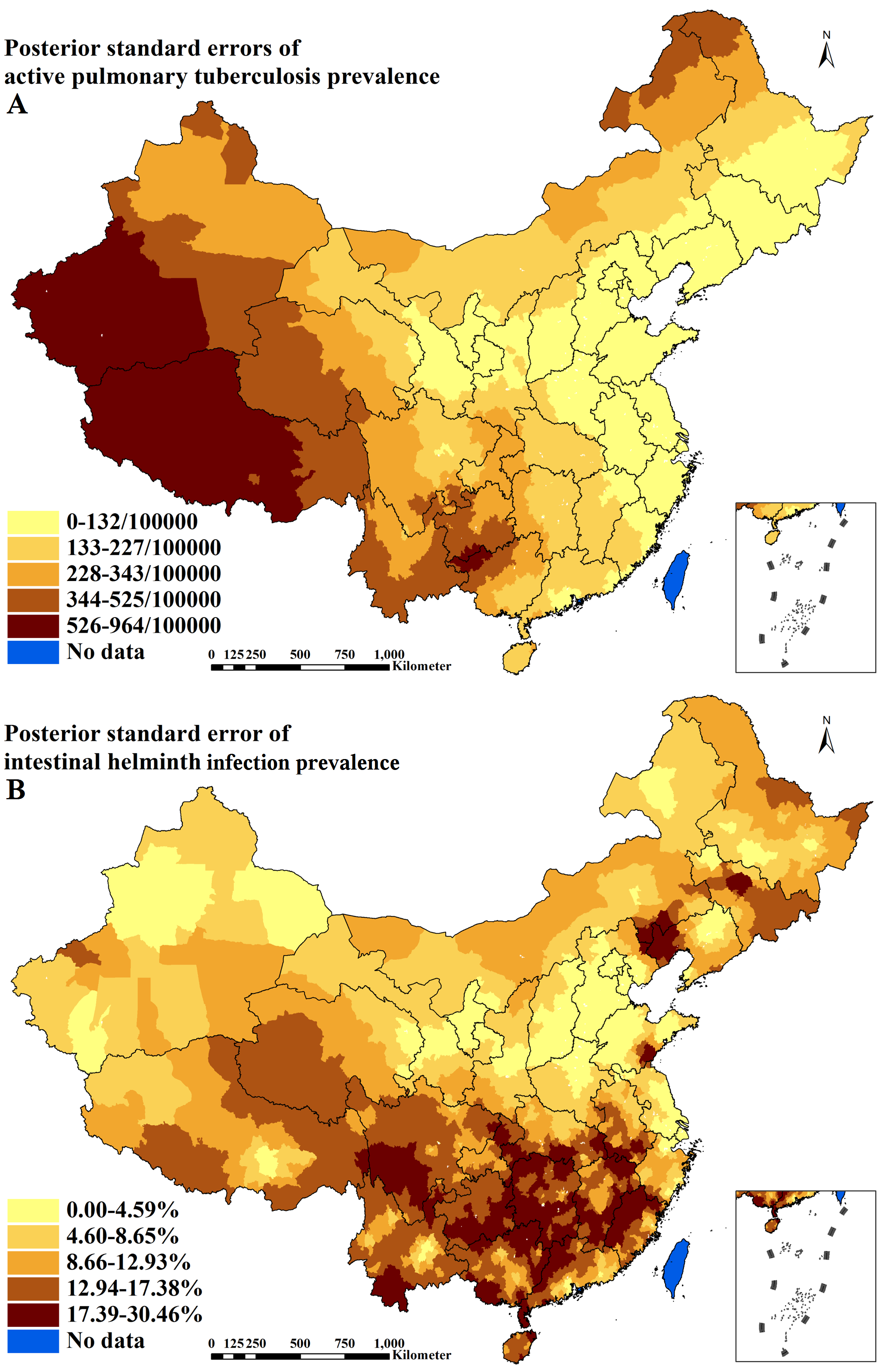

Supplement: S1 Fig — Spatial distributions of posterior standard error of prevalence across P. R. China (A. for active pulmonary tuberculosis; B. for intestinal helminth infection). (TIF) [file pntd.0004580.s003.tif]

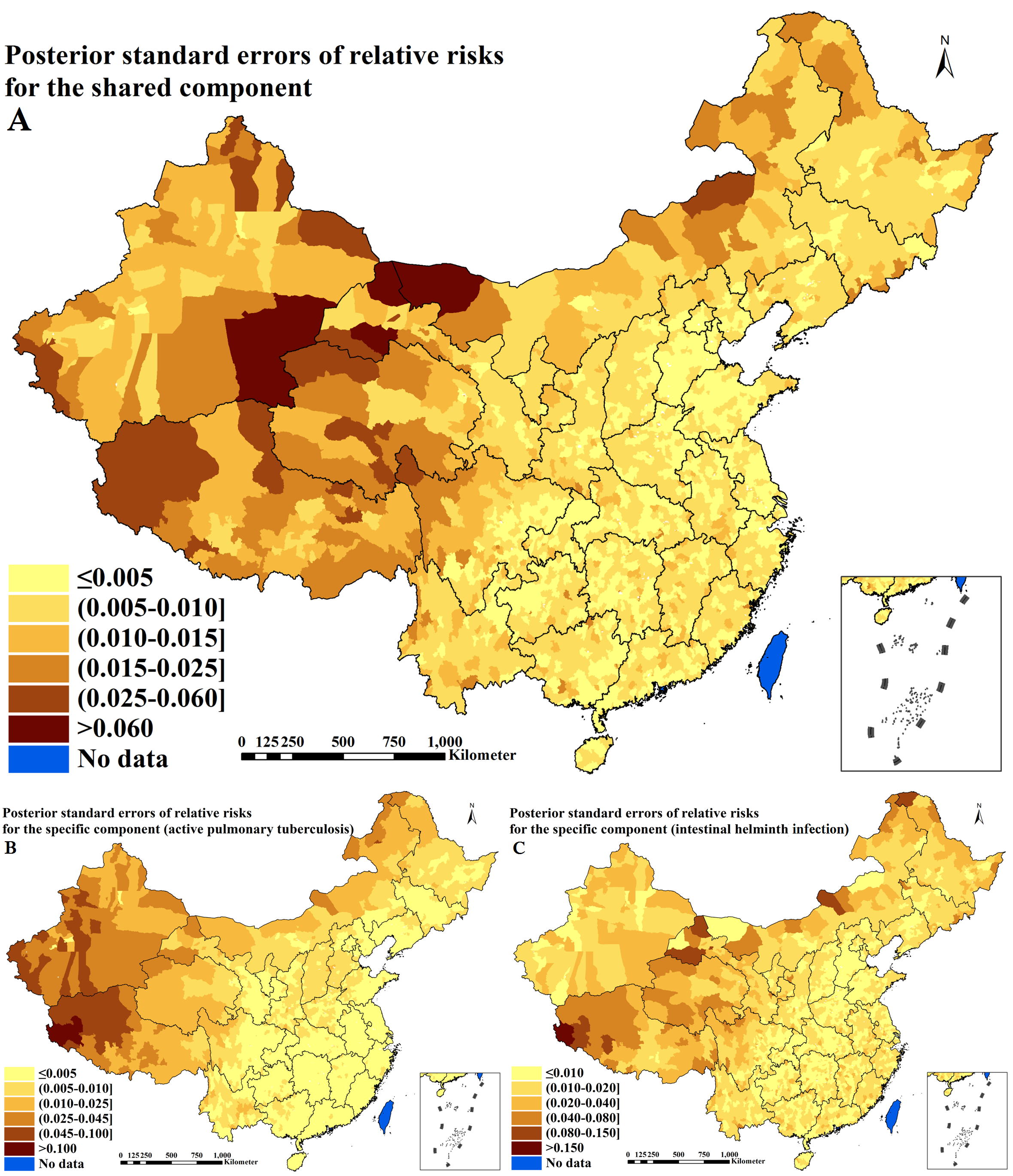

Supplement: S2 Fig — Spatial distributions of posterior standard error of relative risks across P. R. China (A. for the shared component between active pulmonary tuberculosis and intestinal helminth infection; B. for the specific component for active pulmonary tuberculosis; C. for the specific component for intestinal helminth infection). (TIF) [file pntd.0004580.s004.tif]
